# Compassion assessment instruments in palliative care: a scoping review

**DOI:** 10.1186/s12904-025-01870-8

**Published:** 2025-11-26

**Authors:** Carolina Bento Gomes, Patrícia Andreia Weber Marcelino, Manuel Luís Capelas

**Affiliations:** 1 Faculty of Health Sciences and Nursing, Universidade Católica Portuguesa, Lisboa, Portugal; 2 Sesimbra’s town hall, Sesimbra, Portugal; 3 Faculty of Health Sciences and Nursing, Centre for Interdisciplinary Research in Health (CIIS), Portuguese Observatory for Palliative Care (OPCP), Universidade Católica Portuguesa, Lisboa, Portugal

**Keywords:** Compassion, Palliative care, Assessment

## Abstract

**Background:**

Compassion is often described in literature as an indication of quality of care, and it is imperative in the healthcare context, more specifically in the practice of palliative care. This scoping review aimed to identify assessment tools for compassion available in the context of palliative care and describe the psychometric characteristics of the identified assessment tools.

**Methodology:**

A Systematic Review of Literature, a Scoping review. A search was performed in PubMed, CINAHL, MedicLatina, Scopus, Web of Science, and PsycARTICLES computerized databases on March 9, 2022. The participants included were adults, the concept considered was “compassion assessment instruments,” and all studies conducted in the context of palliative care were considered. The protocol was obtained from The Joanna Briggs Institute.

**Results:**

A total of 1371 publications were identified. Of these, only five fulfilled the inclusion criteria. The information was collated in tabular form. Of the five publications selected, only one comprised the original development of the scale, where the other four included studies on palliative care, where the scales were used. It was possible to obtain the original publications in which the scales were developed. Therefore, five instruments for assessing compassion in palliative care were identified: “Patient Assessment of Physician Compassion”; “Sinclair Compassion Questionnaire”; “Compassion from Others Scale”; “Santa Clara Brief Compassion Scale”; “Compassion Scale”.

**Conclusion:**

Only one compassion assessment scale has been developed for palliative care. In addition, of the other four instruments, only one was developed in the healthcare context. This research also indicates that the assessment of compassion in palliative care is recent. The only instrument developed in the context of palliative care was created in 2021, and the first article to describe the assessment of compassion in palliative care was developed in 2018. It also concluded that the assessment of compassion in the field is important, whether from the perspective of the patient or from the perspective of the professional. The instruments have good or excellent internal consistency.

**Supplementary Information:**

The online version contains supplementary material available at 10.1186/s12904-025-01870-8.

## Background

The philosophy of palliative care advocates the promotion of the quality of life of patients and their families, who face problems emerging from an incurable and/or serious illness and have a limited prognosis, through the prevention and resolution of suffering, using early identification and treatment of physical, psychological, social, and spiritual problems [1]. The World Health Organization defines palliative care as care that aims to improve the quality of life of patients and their families, who face problems arising from an incurable and/or serious illness, with a limited prognosis, through the prevention and relief of suffering, using early identification and treatment of physical, psychological, social, and spiritual problems [2].

Compassion is defined in literature as the ability to express empathy, provide emotional support, and show willingness to understand anguish and suffering from others, implying conscious attention and motivation [3]. Doing something for others requires respect, commitment, courage and wisdom [4, 5]. It presents itself as a virtuous response that seeks to address others’ suffering and needs, through understanding and action [6]. In addition to being considered a right of the patient and their family [7], compassion is an essential element in the quality of care provided, which allows health professionals to meet the needs, preferences, and values ​​of their patients and families, enabling individualized care, thus promoting the development of a trusting therapeutic relationship, increasing satisfaction with the care provided, quality of life, and more effective symptom control [8]. Therefore, compassionate care is a fundamental element in improving the quality of services provided in the healthcare system [9]with a positive impact on the experiences of patients and their families [10].

As evident as its importance, the concept “compassion” remains largely theoretical and anecdotal [11]. Only in recent decades, it has come to be seen as a focus of interest in scientific research [12] however, there is international concern regarding the absence of compassion in healthcare. Investigations into how patients experience compassion in a clinical context have only just begun to emerge [5, 12]. This constitutes a serious gap, because consideration of patient and family perspectives are required for a comprehensive understanding of compassion and compassion research [12]. Sinclair et al. [12] conducted a review that included 44 studies, less than one-third of the studies included patients, and the intervention studies included only two compassionate care trials with patients and eight educational programs that aspire to improve compassionate care in clinicians and students [12].

Measuring compassion provides healthcare organizations with the ability to routinely report, monitor, evaluate, and improve compassion across their organization [13]. We know that due to its nature, measuring compassion is a challenge, however, emphasizing patient-centered compassionate healthcare, a psychometrically valid, encompassing instrument for assessing compassion, marked by rigor and flexibility [5]in the context of healthcare and educational institutions is no longer an option, but a necessity [12]. Multiple instruments assess compassion in healthcare. However, a critical and comprehensive analysis of their validity and rigorous determination of psychometric properties are scarce [14, 15].

The objectives of this scoping review are to identify compassion assessment instruments available in the context of palliative care and to describe their psychometric characteristics. Consequently, this scoping review aims to provide health professionals with a global view of the available compassion assessment instruments in palliative care and their respective psychometric properties, thus facilitating the provision of individualized care through the analysis and selection of the assessment instruments that best suit the patient and their current context and situation, with consequent benefits in the provision of care and the experience of the patient and family.

## Methods

In this investigation, the type of study chosen was *scoping review*. This adds to evidence-based practice the examination of a large area and the possible identification of gaps in research and the clarification of concepts. It also clarifies the existing research on the topic and the path of that investigation [16]. The protocol for *scoping reviews* from The Joanna Briggs Institute [15] was used. Available at https://joannabriggs.org/.

The inclusion and exclusion criteria are available in the table below (Table 1).

### Inclusion and exclusion criteria

**Table 1:** Inclusion and exclusion criteria

Inclusion criteria	Participants	Adults (over 18 years old)
	Concept	“Compassion assessment instruments”
	Context	Palliative care units, palliative care practiced outside specific palliative care units, and palliative care provided to patients at the end of life
	Language	English, Portuguese and Spanish
Exclusion criteria	Context	Instruments that assess compassion fatigue and self-compassion
	Type of study	Opinion articles, letters to the editor/director, comments, article analyses, and case studies

### Search strategy

The results were derived from a search in the computerized databases PubMed, CINAHL, MedicLatina, Scopus, Web of Science, and PsycARTICLES, carried out on March 9, 2022. The search strategy with mesh indexing terms, terms searched in the title and abstract, and full electronic search strategy for PubMed are presented in Table 2.

**Table 2:** Search strategy: mesh indexing terms, terms searched in the title and abstract and example: full electronic search strategy for PubMed

Search strategy		
Mesh indexing terms	• Empathy • Compassion • Hospice care • Hospice and palliative care nursing • Terminally ill • Scales • Surveys	• Terminal care • Palliative care • Terminal care • Palliative medicine • Questionnaires • Questionnaires and surveys • Instrument by type
Terms searched in the title and abstract	• Compassion • Empathy • Questionnaire* • Scale* • Assessment • Measure* • Tool* • Test* • Screening	• Instrument • Survey* • Palliative care • Hospice* • Terminal care • End of life • End-of-life • Palliative medicine • Terminally ill
Example: full electronic search strategy for PubMed	(((Empathy[MeSH Terms]) OR (Compassion[Title/Abstract]) OR (Empathy[Title/Abstract])) AND (((((((((Questionnaire*[Title/Abstract]) OR (Scale*[Title/Abstract])) OR (Assessment[Title/Abstract])) OR (Measure*[Title/Abstract])) OR (Tool*[Title/Abstract])) OR (Test*[Title/Abstract])) OR (Screening[Title/Abstract])) OR (Instrument[Title/Abstract])) OR (Questionnaires and Surveys[MeSH Terms]) OR (Survey*[Title/Abstract]))) AND (((((hospice care[MeSH Terms]) OR (Hospice and Palliative Care Nursing[MeSH Terms])) OR (terminally ill[MeSH Terms])) OR (((Palliative Care[MeSH Terms]) OR (Terminal Care[MeSH Terms])) OR (Palliative Medicine[MeSH Terms]))) OR ((((((((Palliative Care[Title/Abstract]) OR (Hospice*[Title/Abstract])) OR (Terminal Care[Title/Abstract])) OR (End of Life[Title/Abstract])) OR (End-of-Life[Title/Abstract])) OR (Palliative Medicine[Title/Abstract])) OR (Terminally ill[Title/Abstract]))))	

A search was also carried out in the following gray literature databases: Open Access Scientific Repository of Portugal and EThOS by the British Library, without success, as no publications that met the inclusion criteria were found.

### Study selection

Initially, a comprehensive search was performed in the platforms and respective databases mentioned above, where the words present in the titles and abstracts of scientific articles and their respective indexing terms were analyzed. A second search was performed using the previously identified indexing terms. At this stage, a search was conducted in the gray literature databases mentioned above. Of the publications that met the search criteria, titles and abstracts were independently analyzed according to the inclusion criteria by two reviewers. The resulting publications were analyzed in full by the authors, according to the inclusion criteria. Minor disagreements between the three authors were resolved.

## Results

### Literature search

**Fig. 1:**
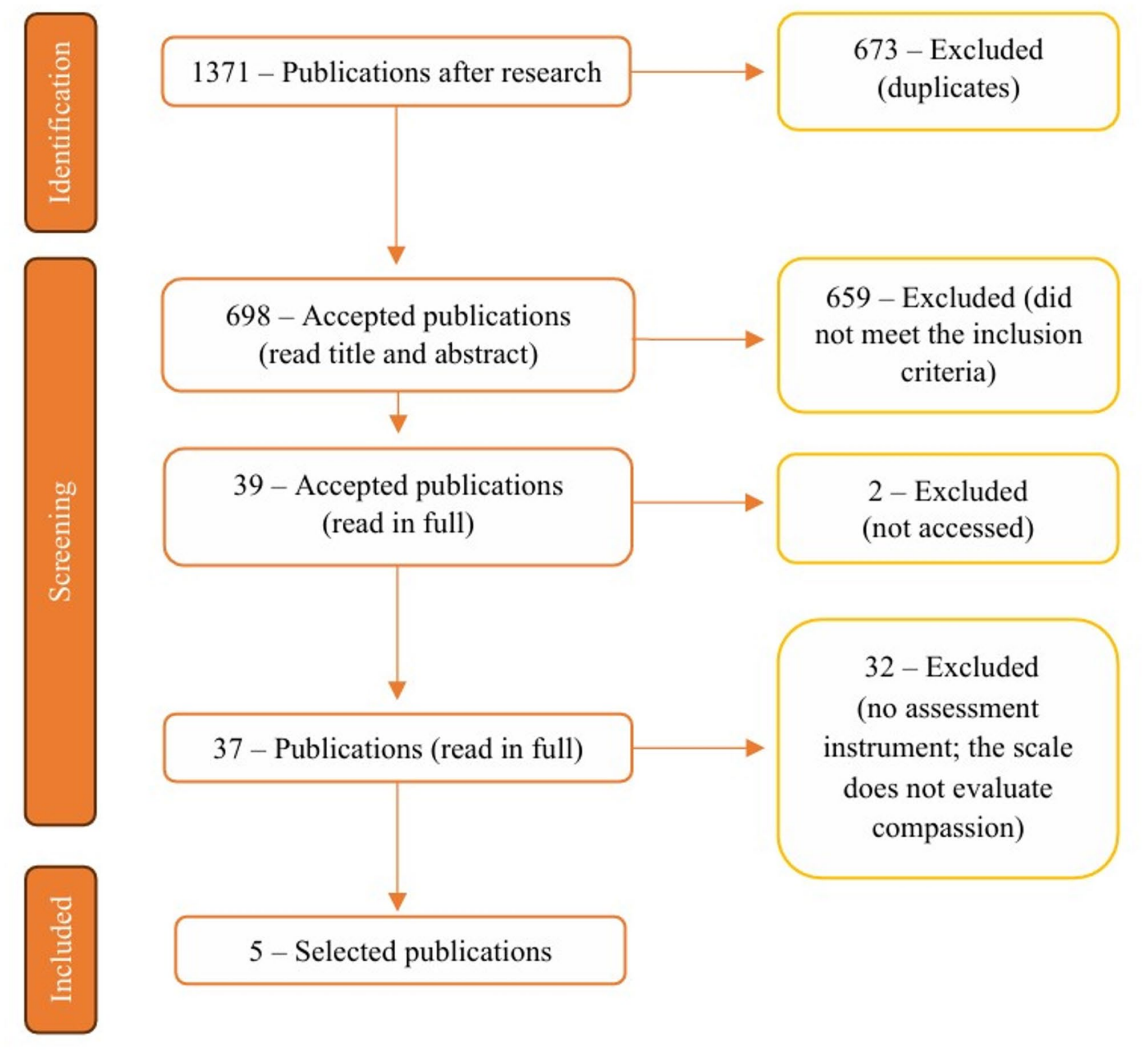
Flow diagram of included publications

The search identified 1371 publications: 511 in PubMed, 275 in CINAHL, one in MedicLatina, 256 in Web of Science, 327 in Scopus and one in PsycoARTICLES. Duplicate publications were excluded (673 publications), leaving 698 potentially relevant publications. Titles and abstracts were screened by two authors, and only 39 publications met the inclusion criteria. Two publications were excluded because they could not be accessed. After a critical review of the 37 publications by the same authors, only five met the inclusion criteria. The othersstudies did not assess an instrument, or the instrument did not evaluate compassion (Fig. 1). Minor disagreements between the three authors were resolved. Of the five publications selected, only one included the original development of the scale. The other four comprised studies within the extent of palliative care, where the scales were used. It was possible to obtain the original publications where the scales were developed. The four publications that described the development of the respective scales did not appear in the research carried out, as they did not meet the inclusion criteria, namely context. Regarding the population, three instruments present university students as participants and one presents healthy women and cancer survivors as participants.

### Identified instruments and their psychometric characteristics

One author extracted data from eligible studies. Of the five selected publications, five different instruments were identified, each publication was developed by different authors. The years of publications ranged from 2018 to 2021. Every study was written in English, three were developed in the United States of America (USA), one in Canada, and the last in the United Kingdom. The population varies among cancer patients, cancer patients and their families, patients with life-limiting conditions, doctors and nurses who work in palliative care, and health professionals who work in palliative care. When it comes to filling out the questionnaire, three scales are filled by patients and the other two are filled by healthcare professionals. To date, only one tool has been developed for use in palliative care.

The instruments analyzed in this study were “Patient Assessment of Physician Compassion” [17]“Sinclair Compassion Questionnaire” [18] (SCQ), “Compassion from Others Scale” [19]“Santa Clara Brief Compassion Scale” [20](SCBCS) and “Compassion Scale” [21]. Information was collated in tabular form, including: title, instrument name and authors’ names, so that professionals can identify the scale; the year of publication, so that professionals can select the most recent literature; language, so that healthcare workers can choose the study in the language they manage; the country where the study was developed, population, and person who fills the instrument, so that professionals can select the articles that better suit their patients (Table 3).

**Table 3:** Information collated from the studies that meet the inclusion criteria, including title, authors names, year of publication, language, country of development of the study, population, scale name, and the person who fills the scale [18, 22–25]

Publication title	Challenging the Status Quo of Physician Attire in the Palliative Care Setting	Development and validation of a patient-reported measure of compassion in healthcare: the Sinclair Compassion Questionnaire	Dispositional mindfulness, self-compassion, and compassion from others as moderators between stress and depression in caregivers of patients with lung cancer	Examining self-care, self-compassion and compassion for others: a cross-sectional survey of palliative care nurses and doctors	Examining the Compassion Status of Healthcare Professionals Working in the Palliative Care Units
Authors	Ahsan Azhar, Kimberson Tanco, Ali Haider, Minjeong Park, Diane Liu, Janet L. Williams, Eduardo Bruera	Shane Sinclair, Thomas F Hack, Cara C MacInnis, Priya Jaggi, Harrison Boss, Susan McClement, Aynharan Sinnarajah, Genevieve Thompson	Chia-Chen Hsieh, Chong‐Jen Yu, Hsiu‐Jung Chen, Yu‐Wen Chen, Nien‐Tzu Chang, Fei‐Hsiu Hsiao	Jason Mills, Timothy Wand, Jennifer A Fraser	Özlem Oruç, Merve Hörmet İğde, Vildan Kocatepe, Dilek Yildirim
Year	2020	2021	2018	2018	2020
Language	English	English	English	English	English
Country of development	USA	Canada	United Kingdom	USA	USA
Population	Cancer patients	Patients with life-limiting conditions	Cancer patients and their families	Doctors and nurses who work in palliative care	Health professionals who work in palliative care
Scale name	Patient Assessment of Physician Compassion [17]	Sinclair Compassion Questionnaire [18]	Compassion from Others Scale [19]	Santa Clara Brief Compassion Scale [20]	Compassion Scale [21]
Filling	Patient	Patient	Patient	Health professionals	Health professionals
Developed in palliative care?	No	Yes	No	No	No

#### Patient assessment of physician compassion

Fogarty et al. [17] developed the “Patient Assessment of Physician Compassion” [17] through the development of stimuli from two videos, with the aim of evaluating patients’ perceptions of the compassion shown by the doctor regarding anxiety, recall of information, treatment decisions and evaluation of characteristics of the doctor himself. In this study, compassion was defined as a supportive concern for the suffering of another, together with an inclination to help, support, or show mercy [6]. The study population comprised 123 female breast cancer survivors and 87 healthy females with no history of breast cancer [6, 17].

Fogarty et al. [17] used five measures. The first was used to evaluate anxiety, it was measured using the State-Trait Anxiety Inventory [17]. The State Anxiety Scale [17] (STAI-S) assesses anxious mood and is commonly used to measure immediate changes in anxiety induced by the experimental procedures. The STAI-S [17] has 20 items answered on a 4-point scale. Individuals were asked to ‘‘indicate how you feel at this moment’’ to statements such as ‘‘I feel calm’’ and ‘‘I am worried”. The answers were as follows: (1) not at all, (2) somewhat, (3) moderately so, and (4) very much so [17]. The second scale was used to evaluate recall of treatment information. The score reflected the quantity of information understood and remembered from the physician’s treatment description in the videotapes. Potential treatment side effects were listed for both low-dose and high-dose chemotherapy treatments, and individuals were asked to recognize the side effects mentioned by the physician for each treatment, and to write in the corresponding probability or chance that the subjects would experience side effects. Questions about the treatment results were also asked. Three types of items comprised the 54-item total information recall score: (1) treatment side effects (29 items), (2) probability of side effects (eight items), and (3) treatment outcome information, such as survival and treatment purpose (17 items). The possible range of the scale is 0−54. The third instrument was intended to assess the extent to which compassion influenced the participants’ perceptions of the general physician attributes. Five pairs of statements were presented using a semantic differential format with a possible range from 0 to 100, (1) ‘‘wants what’s best for the patient’’ as opposed to ‘‘wants what’s best for himself’’, (2) ‘‘encourages patient involvement in treatment decision’’ as opposed to ‘‘discourages patient involvement in treatment decision’’ (3) ‘‘encourages patient’s questions’’ as opposed to ‘‘discourages patient’s questions’’, (4) ‘‘acknowledges patient’s emotions’’ as opposed to ‘‘ignores patient’s emotions’’ and (5) ‘‘cares about the patient’’ as opposed to ‘‘does not care about the patient’’ [17]. The fourth scale was a hypothetical treatment decision. Subjects were asked, ‘‘If you had to choose in the moment between the standard low-dose and high-dose chemotherapy, which treatment would you choose?’’, the participants were given the following response options: ‘‘high-dose chemotherapy,’’ ‘‘low-dose chemotherapy,’’ ‘‘no treatment,’’ or ‘‘I would leave it up to my physician’’ [17].

The “Patient Assessment of Physician Compassion” [17] was created through the development of stimuli from two videos, with the aim of evaluating patients’ perceptions of the compassion shown by the doctor. The videos consisted of staging and dramatizing oncology consultations. In the standard video, the doctor provides information about treatments for metastatic breast cancer and prognosis. In the other video, the doctor provided the same information, but the actors used words and touches to express support, sympathy, and compassion for the patients [6]. Participants rated their physicians’ compassion using a semantic differential format comprising five pairs of physician characteristics. They were warm/cold, pleasant/unpleasant, compassionate/distant, sensitive/insensitive, and affectionate/careless. The antonymous characteristics were presented on a visual analog scale, separated by a 10 cm line [6, 17, 22]. For example, “warm” was at the end of the left side of the line and cold at the end of the right side of the line. Participants were instructed to provide their opinion regarding the doctor within the five items by placing an X on the line closest to the appropriate characteristic. The closer X is to a word, the more this characteristic that doctor exhibits. The distance between X placed by the patient and the closest word was calculated in millimeters so that each item had a possible range of 0 to 10, with a total sum of 0 to 100 [17]. The higher the sum of the final numbers, the greater the perception of compassion shown by the doctor [6, 17]. Azhar et al. [22] described the sum ranging from 0 to 50, maintaining the premise that the higher the sum and the final number, the greater the perception of compassion shown by the doctor [22]. The scale was internally consistent (Cronbach’s alpha coefficient = 0.92), reflecting that it measured a single and cohesive construct [6, 17].

Azhar et al. [22] conducted a study in the context of palliative care, in which the same scale was used. This randomized controlled study aimed to compare the impact of doctor clothing on the perception of compassion, professionalism, and doctor preference among 105 cancer patients. The hypothesis was that patients would perceive doctors in formal clothing as more compassionate than doctors in casual clothing. However, they concluded that the doctor’s attire did not affect cancer patients’ perceptions of the doctor’s level of compassion and professionalism, nor did it influence their preference for their doctor or their confidence in their doctor’s ability to provide care [22].

#### Sinclair compassion questionnaire

Knowing that compassion is the key indicator of quality of care and assuming that, although assessing compassion is a recognized necessity there is a shortage of valid and reliable measures, the “SCQ” [18] was created. This study developed and validated a clinically informed, psychometrically rigorous, patient-reported measure of compassion [18]. Data was collected from participants living with life-limiting illnesses in four care settings (acute, hospice, long-term, and home care). In the first phase, data was analyzed using exploratory factor analysis, and the final items were analyzed using confirmatory factor analysis in the second phase. Thus, an instrument with 15 items and response options based on the Likert scale emerged (1 = strongly disagree, 2 = disagree, 3 = neutral, 4 = agree, and 5 = strongly agree). The “SCQ” [18] has excellent internal consistency (Cronbach’s alpha coefficient 0.96) and test-retest reliability (ranging from 0.74–0.89) [18].

#### Compassion from others scale

This scale emerged as part of a study developed by Gilbert et al. [19] in which the objective was to develop three new compassion assessment measures derived from an evolutionary and motivational approach. The scales assess compassion we experience for others and self-compassion based on the following definition: sensitivity to suffering in oneself and others, with a commitment to trying to alleviate and prevent it. Gilbert et al. [19] in his study explored the relationship between the scales of compassion, self-criticism, depression, anxiety, stress, and well-being. The study population comprised healthy participants from three different countries (the United Kingdom, Portugal and the USA) [19]. The first scale assesses compassion that we experience for others. The authors mention that compassion for others is strongly associated with compassionate love and compassionate goals. In addition, compassion for others, compassionate goals, and compassionate love all had low or non-significant correlations with depression, anxiety, and stress, and only a weak correlation with well-being. However, there is evidence that helping others has positive psychological and physiological benefits. Therefore, the authors defend the importance to distinguish genuine ‘suffering-focused compassion’ from kindness [19]. The second instrument evaluates self-compassion, it is a 13-item scale that consists of an engagement subscale assessing the person’s ability to be sensitive to their own distress/suffering, and an action subscale assessing the person’s ability to be motivated to relieve and prevent distress. Each item is rated on a 10-point Likert scale ranging from 1 (never) to 10 (always). Higher scores on the two subscales indicate higher levels of self‐compassion.

The “Compassion from Others Scale” [19] is the third instrument developed, consisting of 13 items that assess engagement, the ability to be aware of other people’s motivations to be compassionate, and an action subscale, that assesses the individual’s sensitivity to the actions of other people.

The internal consistency of the engagement scale was good (Cronbach’s alpha coefficient = 0.89), and the internal consistency of the action scale was excellent (Cronbach’s alpha coefficient = 0.91) [19].

Hsieh et al. [23] used the “Compassion from Others Scale” [19] in the context of palliative care to identify important protective factors in predicting depressive symptoms in caregivers. In total, 72 respiratory cancer patients and their families participated. The relevant results were the association between depressive symptoms and the domains of full awareness and self-compassionate action, knowing that practicing these last ones has a positive impact on depressive symptoms [23]. The internal consistency in this study was good (engagement subscale with Cronbach’s alpha coefficient = 0.65, action subscale with Cronbach’s alpha coefficient = 0.90, and total score Cronbach’s alpha coefficient = 0.85) [23].

#### Compassion scale

Pommier [21] develpoed the present instrument based on a construct that, to empirically examine compassion, it is necessary to develop an appropriate scale to measure the concept. Once the scale is in place, it opens the possibility of discussion and additional empirical understanding of the construct in science. The author also mentions that although there are a series of studies that attempt to study compassion, they do so without investigating it and using an appropriate scale [21]. The participants at the root of the instrument were 439 healthy university students (153 males and 286 females) [21]. The scale consists of 24 items and the scores are classified using a five-point Likert scale and it has six subscales: kindness (items 6, 8, 16, 24), indifference (items 2, 12, 14, 18), common humanity (items 11, 15, 17, 20), separation (items 3, 5, 10, 22), mindfulness (items 4, 9, 13, 21), and non-involvement (items 1, 7, 19, 23). The indifference, separation, and non-involvement subscales are reverse scored. The score was calculated by obtaining the total average score. As the total score obtained on the scale increases, so does the level of compassion [21, 25]. This instrument has good internal consistency (Cronbach’s alpha coefficient = 0.90), good content validity, and a high intercorrelation between the six subscales [25].

Oruç et al. [25] used the present scale in the context of palliative care in a study that aimed to examine compassion practiced by health professionals, who worked in palliative care units, from the perspective of the professionals themselves. The sample consisted of 81 healthcare professionals working in three hospitals in Istanbul. The study was conducted based on the construct that many factors affect feelings of compassion from health professionals who work in palliative care, especially with end-of-life patients. It is important for them to understand the factors that can affect their sense of compassion and control their negative characteristics [25].

#### Santa Clara brief compassion scale

The present scale was developed using the abbreviation of the “Sprecher and Fehr’s Compassionate Love Scale” [26]. Hwang et al. [20] developed the “SCBCS” [20]. It results from questionnaires administered to 223 students at the University of Santa Clara (167 females and 56 males). Its small size makes it easy to use, particularly for large epidemiological studies. It consists of five items, where responses have a numerical range from 1 to 7 (“not true” to “completely true”, respectively). It has good internal consistency (Cronbach’s alpha coefficient = 0.90) [20] and good content validity [24].

It was used in the context of palliative care by Jason Mills et al. [24] in a study that examined the levels and relationships between self-care capacity, self-compassion, and compassion among palliative care nurses and doctors. A total of 369 Australian professionals participated in this study, having concluded that levels of compassion, self-compassion, and self-care capacity vary among each individual [24].

Therefore, the main results show that there is only one instrument that evaluates compassion developed in a palliative care setting. All instruments show good or excellent internal consistency, and only three describe their validity.

## Discussion

Compassion is described in the literature as a right of the patient and their family [14] which facilitates the development of a trusting therapeutic relationship with a consequent increase in satisfaction with the care provided, and the other’s quality of life. It is also seen as an essential element in the quality of care provided, making it easier for healthcare professionals to meet the needs, preferences, and values ​​of their patients and families, thus focusing care on the person [14, 27]. Despite all efforts on the part of the scientific community, and evidence regarding the importance and need for compassion, its absence is one of the main contributing factors to physical and emotional neglect in healthcare [8].

In this *scoping review*, compassion assessment instruments available in the context of palliative care were identified (“Patient Assessment of Physician Compassion” [17]“SCQ” [18]“Compassion from Others Scale” [19]“SCBCS” [20] and “Compassion Scale” [21]). However, only one was developed in the context of palliative care (“SCQ” [18]), one in the healthcare context (“Patient Assessment of Physician Compassion” [17]) and three in the academic area (“Compassion from Others Scale” [19]“SCBCS” [20] and “Compassion Scale” [21]). Psychometric characteristics of the scales have also been described. This allows health professionals to have a global view of the available assessment instruments and their psychometric properties, thus facilitating the provision of individualized care through the analysis and selection of an assessment instrument that best suits their context and current situation, with consequent benefits in the provision of care and experience to the patient and family.

The literature states that, although there are multiple instruments that assess compassion in healthcare, critical and comprehensive analyses of their validity and rigorous are scarce [14, 15]. Regarding the present investigation, all instruments describe their fidelity (three have good internal consistency (“Compassion from Others Scale” [19]“SCBCS” [20] and “Compassion Scale” [21]), and the other two have excellent internal consistency (“Patient Assessment of Physician Compassion” [17]“SCQ” [18])), however, regarding validity, only three studies describe it (“SCQ” [18]“SCBCS” [20] and “Compassion Scale” [21]).

We also know that compassion has only been evaluated from the perspective of healthcare professionals for a long time. Sinclair et al. [12] conducted a review that included 44 studies, less than one-third of the studies included patients, and the intervention studies included only two compassionate care trials with patients. This study recognizes the limited empirical understanding of compassion in healthcare and focuses on the lack of patient and family voices in compassion investigations [12]. However, publications investigating how patients and their families experience compassion in healthcare are beginning to emerge [5]. Regarding the research carried out, two of the instruments were developed from the perspectives of compassion practitioners (“Compassion Scale” [21] and “SCBCS” [20]), while the other three were developed from the perspective of compassion recipients (“Patient Assessment of Physician Compassion” [17]“SCQ” [18]and “Compassion from Others Scale” [19]).

Additionally, although the compassion construct has been discussed for several centuries the related literature is very recent [28] which was verified in the present study where the assessment instruments were developed between 1999 and 2021.

The diversity of the context in which the instruments were developed allows us to conclude that, although compassion is an indicator of care and inherent to the provision of palliative care, its evaluation in this context remains scarce, as only one tool was developed in the context of palliative care (“SCQ” [18]) and of the other four resulting scales, only one was developed in the healthcare context (“Patient Assessment of Physician Compassion” [17]). However, all scales have good or excellent internal consistency.

### Limitations

The intensive description of the methodology, the process of extracting results, and the use of two independent reviewers made it possible to reduce the limitations and biases inherent to all research processes, however, they were not eliminated entirely. The first limitation is the diversity of the databases consulted, making the investigation dependent on the existence of publications that meet the inclusion criteria, leaving doubts about whether the research process was sufficiently comprehensive. Another important limitation to mention is the limitation of the language of the publications. The fact that only studies written in English, Portuguese, and Spanish were included may have excluded important publications.

## Conclusion

This review searched for instruments that assess compassion in palliative care and described its psychometric characteristics. The diversity of the context in which the instruments were developed allows us to conclude that, although compassion is an indicator of care and the inherent provision of palliative care, its assessment in this context is still scarce, as there is only one compassion assessment instrument available in the context of palliative care. In Addition, from the other four scales, only one has been developed in the healthcare context. The research also indicates that, despite compassion being mentioned in literature for a long time, its assessment in palliative care is recent. The only instrument developed in the context of palliative care was created in 2021, and the first article describing the assessment of compassion in palliative care was published in 2018. When it comes to measuring compassion practiced by the physician, three instruments were from the patient’s optic, and the remaining two from the perspective of healthcare professionals. It was concluded that the assessment of compassion in the field is important, whether from the optic of the patient or from the perspective of the professional. In the future, it would be important to develop instruments for assessing compassion in palliative care that are reliable and valid by experts in the matter, as there is currently only one study, and increase the assessment of compassion in palliative care, as there are currently four studies.

## Supplementary Information

Additional file 1. Index terms used in the search conducted in computerized databases PubMed, CINAHL, MedicLatina, Scopus, Web of Science and PsycARTICLES, carried out on March 9, 2022.

Additional file 2. PRISMA Check list for Scoping Reviews, was filled out according to the present scoping review.

## Data Availability

No datasets were generated or analysed during the current study.

## Abbreviations

SCQ: Sinclair Compassion Questioner
USA: United States of America
SCBCS: Santa Clara Brief Compassion Scale
STAI: S–State–Trait Anxiety Inventory Scale
