# Supplementary material for: Compassion assessment instruments in palliative care: a scoping review
Source: BMC Palliat Care. 2025 Nov 26;25:1. doi: 10.1186/s12904-025-01870-8 (PMC12763884; doi:10.1186/s12904-025-01870-8)
Supplement: Supplementary file 1 — Additional file 1. Index terms used in the search conducted in computerized databases PubMed, CINAHL, MedicLatina, Scopus, Web of Science and PsycARTICLES, carried out on March 9, 2022. [file 12904_2025_1870_MOESM1_ESM.docx]

**Additional File 1: Index terms used in research**

Search in the computerized databases PubMed, CINAHL, MedicLatina, Scopus, Web of Science and PsycARTICLES, was carried out on March 9, 2022, with the index terms below.

- PubMed: (((Empathy[MeSH Terms]) OR (Compassion[Title/Abstract]) OR (Empathy[Title/Abstract])) AND (((((((((Questionnaire*[Title/Abstract]) OR (Scale*[Title/Abstract])) OR (Assessment[Title/Abstract])) OR (Measure*[Title/Abstract])) OR (Tool*[Title/Abstract])) OR (Test*[Title/Abstract])) OR (Screening[Title/Abstract])) OR (Instrument[Title/Abstract])) OR (Questionnaires and Surveys[MeSH Terms]) OR (Survey*[Title/Abstract]))) AND (((((hospice care[MeSH Terms]) OR (Hospice and Palliative Care Nursing[MeSH Terms])) OR (terminally ill[MeSH Terms])) OR (((Palliative Care[MeSH Terms]) OR (Terminal Care[MeSH Terms])) OR (Palliative Medicine[MeSH Terms]))) OR ((((((((Palliative Care[Title/Abstract]) OR (Hospice*[Title/Abstract])) OR (Terminal Care[Title/Abstract])) OR (End of Life[Title/Abstract])) OR (End-of-Life[Title/Abstract])) OR (Palliative Medicine[Title/Abstract])) OR (Terminally ill[Title/Abstract])))) Filters: English, Portuguese, Spanish
- CINAHL: ( MH compassion OR ( TI Compassion OR AB Compassion ) OR TI empathy OR AB empathy ) AND ( ( MH Questionnaires OR MH Instrument by Type OR MH Scales OR MH Surveys ) OR ( TI Questionnaire* OR AB Questionnaire* OR TI Scale* OR AB Scale* OR TI Assessment OR AB Assessment OR TI Measure* OR AB Measure* OR TI Tool* OR AB Tool* OR TI Test* OR AB Test* OR AB survey* OR TI survey* OR TI Screening OR AB Screening OR TI Instrument OR AB Instrument ) ) AND ( ( MH palliative care OR MH Hospice Care OR MH terminal care OR MH Palliative Medicine OR MH ( Hospice and Palliative Care Nursing ) OR MH Terminally ill ) OR ( TI Palliative Care OR AB Palliative Care OR TI Hospice* OR AB Hospice* OR TI Terminal Care OR AB Terminal Care OR TI End of Life OR AB End of Life OR TI Endof-Life OR AB End-of-Life OR TI palliative medicine OR AB Palliative Medicine OR TI terminally ill OR AB terminally ill ) )
- MedicLatina: ( ( ( TI Compassion OR AB Compassion ) OR TI empathy OR AB empathy ) AND ( TI Questionnaire* OR AB Questionnaire* OR TI Scale* OR AB Scale* OR TI Assessment OR AB Assessment OR TI Measure* OR AB Measure* OR TI Tool* OR AB Tool* OR TI Test* OR AB Test* OR AB survey* OR TI survey* OR TI Screening OR AB Screening OR TI Instrument OR AB Instrument ) ) AND ( TI Palliative Care OR AB Palliative Care OR TI Hospice* OR AB Hospice* OR TI Terminal Care OR AB Terminal Care OR TI End of Life OR AB End of Life OR TI End-of-Life OR AB End-of-Life OR TI palliative medicine OR AB Palliative Medicine OR TI terminally ill OR AB terminally ill )
- Scopus:( ( TITLE ( compassion ) OR ABS ( compassion ) OR TITLE ( empat hy ) OR ABS ( empathy ) ) ) AND ( ( TITLE ( questionnaire* ) OR ABS ( q uestionnaire* ) OR TITLE ( scale* ) OR ABS ( scale* ) OR TITLE ( assess ment ) OR ABS ( assessment ) OR TITLE ( measure* ) OR ABS ( measure* ) OR TITLE ( tool* ) OR ABS ( tool* ) OR TITLE ( test* ) OR ABS ( test * ) OR TITLE ( survey* ) OR ABS ( survey* ) OR TITLE ( screening ) OR ABS ( screening ) OR TITLE ( instrument ) OR ABS ( instrument ) ) ) AND ( ( TITLE ( palliative W/1 care ) OR ABS ( palliative W/1 care ) OR TITL E ( hospice* ) OR ABS ( hospice* ) OR TITLE ( "Terminal care" ) OR ABS ( "Terminal care" ) OR TITLE ( "End of Life" ) OR ABS ( "End of Life" ) OR TITLE ( end-of-life ) OR ABS ( endoflife ) OR TITLE ( palliative W/1 medicine ) OR ABS ( palliative W/1 medicine ) OR ABS ( "Terminally ill" ) OR TITLE ( "Terminally ill" ) ) )
- Web of Science: ((ALL=( (((TI=(compassion)) OR AB=(compassion)) OR TI=(empathy)) OR AB=(empathy))) AND ALL=((((((((((((((((((TI=(Questionnaire* )) OR AB=(Questionnaire* )) OR AB=(Scale* )) OR AB=(Assessment)) OR AB=(Measure*)) OR AB=(Tool*)) OR AB=(Test* )) OR AB=(survey*)) OR AB=(Screening )) OR AB=(Instrument )) OR TI=(Scale* )) OR TI=(Assessment)) OR TI=(Measure*)) OR TI=(Tool*)) OR TI=(Test*)) OR TI=(survey*)) OR TI=(Screening)) OR TI=(Instrument))) AND ALL=((((((((((((TI=(Palliative NEAR/1 Care)) OR TI=(Hospice*)) OR TI=("Terminal care")) OR TI=("End of Life" )) OR TI=(End-of-Life )) OR TI=(Palliative NEAR/1 Medicine )) OR AB=(Palliative NEAR/1 Medicine )) OR AB=(End-of-Life )) OR AB=("End of Life" )) OR AB=("Terminal care")) OR AB=(Hospice*)) OR AB=(Palliative NEAR/1 Care ) OR (TI=("Terminally ill")) OR AB=("Terminally ill"))
- PsycARTICLES: ( ( TI empathy OR AB empathy OR TI Compassion OR AB Compassion ) OR MJ empathy ) AND ( ( MJ measurement OR MJ tool use OR MJ screening ) OR ( ( TI Questionnaire* OR AB Questionnaire* OR TI Scale* OR AB Scale* OR TI Assessment OR AB Assessment OR TI Measure* OR AB Measure* OR TI Tool* OR AB Tool* OR TI Test* OR AB Test* ) OR ( TI survey* OR AB survey* OR TI Screening OR AB Screening) ) AND ( ( ( TI terminal care OR AB Terminal care OR TI Hospice* OR AB Hospice* OR TI Palliative Care OR AB Palliative Care ) OR TI Palliative Medicine OR AB palliative medicine OR TI End-of-Life OR AB End-of-Life OR TI End of Life OR AB End of Life ) ) OR ( MJ Palliative Care OR MJ Terminally Ill Patients ) )
